# Overexpression of Mitochondrial Sirtuins Alters Glycolysis and Mitochondrial Function in HEK293 Cells

**DOI:** 10.1371/journal.pone.0106028

**Published:** 2014-08-28

**Authors:** Michelle Barbi de Moura, Radha Uppala, Yuxun Zhang, Bennett Van Houten, Eric S. Goetzman

**Affiliations:** 1 Department of Pharmacology and Chemical Biology, University of Pittsburgh, Pittsburgh, Pennsylvania, United States of America; 2 University of Pittsburgh Cancer Institute, Pittsburgh, Pennsylvania, United States of America; 3 Division of Medical Genetics, Department of Pediatrics, Children’s Hospital of Pittsburgh of The University of Pittsburgh Medical Center, Pittsburgh, Pennsylvania, United States of America; Universidad Pablo de Olavide, Centro Andaluz de Biología del Desarrollo-CSIC, Spain

## Abstract

SIRT3, SIRT4, and SIRT5 are mitochondrial deacylases that impact multiple facets of energy metabolism and mitochondrial function. SIRT3 activates several mitochondrial enzymes, SIRT4 represses its targets, and SIRT5 has been shown to both activate and repress mitochondrial enzymes. To gain insight into the relative effects of the mitochondrial sirtuins in governing mitochondrial energy metabolism, SIRT3, SIRT4, and SIRT5 overexpressing HEK293 cells were directly compared. When grown under standard cell culture conditions (25 mM glucose) all three sirtuins induced increases in mitochondrial respiration, glycolysis, and glucose oxidation, but with no change in growth rate or in steady-state ATP concentration. Increased proton leak, as evidenced by oxygen consumption in the presence of oligomycin, appeared to explain much of the increase in basal oxygen utilization. Growth in 5 mM glucose normalized the elevations in basal oxygen consumption, proton leak, and glycolysis in all sirtuin over-expressing cells. While the above effects were common to all three mitochondrial sirtuins, some differences between the SIRT3, SIRT4, and SIRT5 expressing cells were noted. Only SIRT3 overexpression affected fatty acid metabolism, and only SIRT4 overexpression altered superoxide levels and mitochondrial membrane potential. We conclude that all three mitochondrial sirtuins can promote increased mitochondrial respiration and cellular metabolism. SIRT3, SIRT4, and SIRT5 appear to respond to excess glucose by inducing a coordinated increase of glycolysis and respiration, with the excess energy dissipated via proton leak.

## Introduction

Mitochondrial energy metabolism is finely tuned to match energy production with demand. Mitochondria are able to adapt to changes in energy demand by changing in number and size, as well as by changing substrate preference and flux [1]–[3]. Post-translational modifications to the bioenergetic machinery, most notably through lysine acylation, are emerging as important regulators of mitochondrial function [4]. The acylation of mitochondrial proteins may involve both enzymatic and non-enzymatic mechanisms [5]–[7]. Their reversal is catalyzed by a family of NAD-dependent lysine deacylases known as the sirtuins (SIRTs). Humans have seven SIRT enzymes, three of which localize primarily to mitochondria (SIRT3, SIRT4, and SIRT5). [8]. Of the three mitochondrial-localized sirtuins, SIRT3 is the best characterized, with 15+ substrate proteins reported [4]. In general, enzymatic activity of SIRT3 substrate proteins is increased following deacetylation, suggesting that protein acetylation suppresses mitochondrial function while SIRT3 restores/activates function [4]. SIRT3 is now recognized as the dominant mitochondrial deacetylase and SIRT3 knockout mice show dramatic increases in mitochondrial protein acetylation. SIRT5, on the other hand, has been shown to prefer malonyllysine, succinyllysine, and glutaryllysine as substrates over acetyllysine [9], [10]. SIRT5 desuccinylation has been reported to reduce the activity of pyruvate dehydrogenase and succinate dehydrogenase [11], but increase the activity of 3-hydroxy-3-methylglutaryl-CoA synthase 2 [12]. In contrast to SIRT3 and SIRT5, SIRT4 was originally reported to be a lysine ADP-ribosyltransferase rather than a deacylase [13], but that has been challenged [14]. Recently, SIRT4 was shown to deacetylate and thereby repress mitochondrial malonyl-CoA decarboxylase [14], [15]. SIRT4 has been proposed to inhibit both glutamine and fatty acid oxidation.

The objective of the current study was to directly compare the effects of SIRT3, SIRT4, and SIRT5 on global mitochondrial function and energy metabolism using an over-expression system in HEK293 cells.

## Materials and Methods

### Cell lines and culture

HEK293 cells obtained from ATCC and were cultured in DMEM with 10% fetal bovine serum at 37°C and 5% CO_2_. In some experiments DMEM with 5 mM glucose was used. Cells were stably transfected with pcDNA3.1 vectors bearing HA-tagged SIRT3, SIRT4, or SIRT5 which were kind gifts of Dr. Eric Verdin. The control cell line was stably transfected with the empty vector.

### Western blotting

Antibodies used were: anti-HA (Abcam, 1∶2000), anti-human SIRT3 (Epitomics, 1∶500), anti-human SIRT4 (Antibodies-Online, 1∶500), anti-human SIRT5 (Abcam, 1∶5000), anti-cytochrome-C (Pierce, 1∶500) anti-very long-chain acyl-CoA dehydrogenase (1∶1000; gift of Dr. Jerry Vockley), and respiratory chain antibody cocktail (1∶1000; Mitosciences, Eugene, OR). Cells were lysed in RIPA buffer and the homogenates were cleared by centrifugation and analyzed for protein concentration in triplicate using the Bradford method (Bio-Rad Hercules, CA). Lysates were electrophoresed and transferred to nitrocellulose membranes using the Bio-Rad Criterion apparatus. For western blotting of cell fractions, cell pellets were gently dispersed in 250 mM sucrose, 1 mM EDTA, 10 mM Tris, pH 7.4. The cell suspensions were lysed mechanically by 20 passes through a cell homogenizer (Isobiotech, Heidelberg, Germany) using 10 µM clearance. Unbroken cells and nuclei were removed and discarded by centrifugation at 1,000×g for 10 minutes. Mitochondria were collected by centrifuging the supernatant at 12,000×g for 15 minutes. The supernatant was taken as the cytosolic fraction and the pellet as the mitochondrial fraction.

### Extracellular flux analysis

The oxygen consumption rate and extracellular acidification rate were measured as we have described using a Seahorse XF24 Extracellular Flux Analyzer (Seahorse Bioscience, North Billerica, MA) [16]–[18]. The four cell lines were measured simultaneously with quadruplicate wells per cell line. Then the entire experiment was repeated. To ensure equal cell numbers across the four cell lines, cells were seeded in XF24 cell culture plates coated with Cell-Tak (BD Biosciences, San Jose, CA) at 4×10^4^ cells/well and incubated at 37°C for one hour in unbuffered, serum-free DMEM media prior to analysis. Bioenergetic profiling was performed by monitoring basal oxygen consumption for 30 minutes followed by the sequential injection of the following inhibitors, with the final concentrations indicated in parentheses: oligomycin (1 µM), cyanide *p*-trifluoromethoxy-phenylhydrazone (FCCP; 300 nM), 2-deoxyglucose (100 mM), and rotenone (1 µM). Basal oxygen consumption, proton leak, oligomycin-stimulated extracellular acidification, and the oxygen consumption/extracellular acidification ratios were calculated from the primary data as described in the text.

### ATP, Mitochondrial membrane potential, superoxide, and NAD^+^

Steady-state ATP levels were measured using a luminescence-based ATP detection assay (ATPlite PerkinElmer Inc., Waltham, MA). Briefly, 4×10^4^ cells/well were seeded in a 96-well black plate and treated for 45 minutes with the indicated compounds (100 µM etomoxir, 1 µM oligomycin, 100 mM 2-deoxyglucose, or combinations thereof). Cells were lysed, substrate solution was added, and luminescence was measured using a Biotek Synergy 2 plate reader (Winooski, VT).

Mitochondrial membrane potential and superoxide were determined by incubating cells for 20 min at 37°C in 20 nM of tetramethylrhodamine methyl ester (TMRM; Life Technologies, Carlsbad, CA) or 2.5 µM of MitoSox (Life Technologies, Carlsbad, CA), respectively [19]. Six wells of cells per cell line were analyzed, and the entire experiment repeated on another batch of cells. Fluorescence intensity was analyzed using a CyAn ADP Analyzer (Beckman Coulter, Brea, CA). For NAD+, mitochondria were isolated from confluent T75 flasks of 293 cells as described under western blotting, lysed in extraction buffer from a commercial NADH/NAD determination kit (BioVision, Milpitas, CA), and NAD was measured following the manufacturer’s protocol. The assay was done on three separate mitochondrial isolations (three T75 flasks of each cell line).

### Substrate metabolism

All four cell lines were assayed simultaneously in quadruplicate in 24-well plates, with rates of metabolism normalized to cellular protein content. For glucose oxidation, cells were incubated at 37°C with uniformly labeled ^14^C-glucose in serum-free DMEM (37 kBq per ml) containing 25 mM glucose for one hour in a sealed plate apparatus [20]. Perchloric acid was introduced needlewise through the rubber gasket to acidify the media, and the plate was further incubated for another hour at 37°C to trap ^14^C-CO_2_. Palmitate oxidation was also performed in the sealed trapping plate. Cells were starved in phosphate buffered saline for 30 minutes prior to the assay. ^14^C-palmitate conjugated to bovine serum albumin was added to the cells (125 µM final) in phosphate buffered saline supplemented with 1 mM carnitine. Trapping of ^14^C-CO_2_ was performed using same method as for ^14^C-glucose. ^14^C-acetate metabolism was used to follow fatty acid synthesis. Cells growing in complete DMEM were spiked with 37 KBq per ml of ^14^C-acetate and incubated overnight. Then, the media was removed and cells were lysed and subjected to total lipid extraction by the method of Bligh and Dyer [21].

### Statistical evaluation

Sirtuin overexpressing cell lines were statistically compared to vector-only control cells using Student’s t-test. All data are presented as means with standard deviations.

## Results

### Expression and localization of HA-tagged sirtuins in HEK293 cells

**Figure 1:**
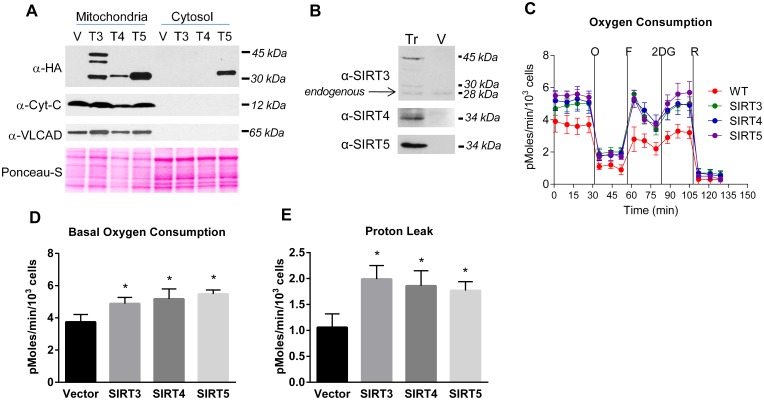
Effect of mitochondrial sirtuin expression on the cellular oxygen consumption rate. (A) HEK293 cells stably transfected with either HA-tagged SIRT3, SIRT4, SIRT5, or the empty plasmid vector (V) were fractionated into mitochondria and cytosol and subjected to western blotting with anti-HA to visualize the tagged sirtuins. Anti-cytochrome-C (Cyt-C) and anti-very long-chain acyl-CoA dehydrogenase (VLCAD) antibodies were used as markers to demonstrate that the mitochondria were intact. Ponceau-S staining served as a loading control. (B) Anti-sirtuin western blotting of stably transfected (Tr) HEK293 cell lines versus vector (V) transfected control cells picks up the same isoforms as anti-HA blotting in panel A, and also visualizes weak expression of endogenous SIRT3 (indicated with arrow). No endogenous SIRT4 or SIRT5 were detected under the conditions used. (C) Equal numbers of the stably transfected HEK293 cells were subjected to oxygen consumption measurements in a Seahorse XF24 extracellular flux analyzer, with sequential additions of the metabolic inhibitors/activators oligomycin (O), FCCP (F), 2-deoxyglucose (2DG), and rotenone (R). The measurements were done in quadruplicate wells of cells. The experiment was repeated with similar results. Oxygen consumption data over the first 30 minutes was averaged to yield the basal oxygen consumption parameter (D). Proton leak (E), defined as oligomycin-insensitive oxygen consumption was calculated by averaging the data over the second 30 minute period (following oligomycin injection). All graphs depict means and standard deviations, and *P<0.01.

HEK293 and HEK293T cell lines have previously been used successfully to study SIRT3, SIRT4, and SIRT5 function, and therefore we chose HEK293 as the cell line in which to directly compare the mitochondrial sirtuins [11], [13], [22], [23]. HEK293 cells were stably transfected with HA-tagged SIRT3, SIRT4, or SIRT5. Western blotting with anti-HA antibody revealed three SIRT3 isoforms, consistent with previous reports [24], and only one isoform for SIRT4 and SIRT5 (Figure 1A). Anti-SIRT3 antibody detected a total of four bands–the same three HA-tagged isoforms in addition to the endogenous untagged 28 kDa protein, which is equivalent to the smallest of the HA-tagged forms but runs faster due to the absence of the tag (indicated by arrow in Figure 1B). In contrast, no endogenous SIRT4 or SIRT5 were detected in HEK293 cells (Figure 1B). To determine intracellular localization of the HA-tagged sirtuins, mitochondrial and cytosolic fractions were western blotted with anti-HA antibody. All three of the SIRT3 isoforms localized to mitochondria (Figure 1A). SIRT4 also localized strictly to mitochondria. SIRT5 was detected in both mitochondria and the cytosol, in confirmation of previous reports [25]. Anti-cytochrome C and anti-very long-chain acyl-CoA dehydrogenase antibodies were used to demonstrate that the cytosolic fraction was not contaminated with either intra-mitochondrial membrane proteins (cytochrome C) or mitochondrial matrix proteins (very long-chain acyl-CoA dehydrogenase).

### Overexpression of mitochondrial sirtuins increases mitochondrial respiration

The effects of SIRT3, SIRT4, and SIRT5 overexpression on overall mitochondrial function were compared using a Seahorse XF24 extracellular flux analyzer to measure the oxygen consumption rate, an indicator of oxidative phosphorylation, in the presence of a series of metabolic inhibitors and uncoupling agents (Figure 1C). Basal oxygen consumption was significantly increased in all three sirtuin-overexpressing cell lines compared to vector-transfected control cells (Figure 1C, D). The rate of oligomycin-insensitive oxygen consumption, which reflects proton leakage across the inner mitochondrial membrane [26], was significantly higher in all three sirtuin-overexpressing cell lines (Figure 1E). After oligomycin, cells were injected with FCCP which permeabilizes the inner mitochondrial membrane and induces maximal, uncoupled respiration. The response to FCCP, defined as the percent increase over basal oxygen consumption, did not significantly differ among cell lines. Thus, while basal oxygen consumption is higher, much of this appears to be due to increased proton leak, and the spare respiratory capacity is not increased by sirtuin overexpression.

**Figure 2:**
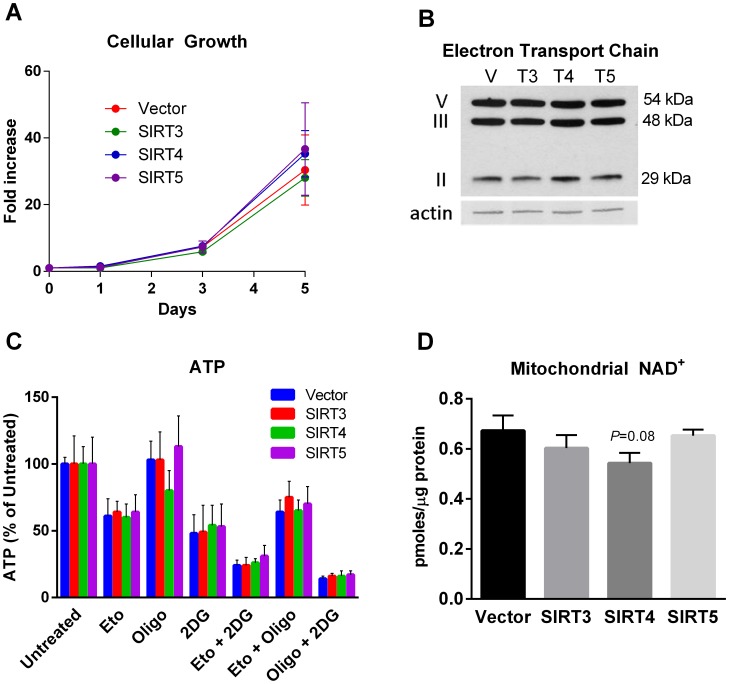
Sirtuin expression in HEK293 does not affect (A) the rate of cellular growth; (B) mitochondrial mass as judged by western blotting of electron transport chain components; (C)steady-state ATP under basal conditions or after the addition of the metabolic inhibitors etomoxir (Eto, 100 µM), oligomycin (oligo, 1 µM), 2-deoxyglucose (2DG, 100 mM), or combinations thereof; or (D) intramitochondrial NAD^+^. Growth was measured in quadruplicate wells in two separate experiments which were averaged. ATP was measured in triplicate wells containing equal numbers of cells in two separate experiments which were averaged. NAD+ was measured in three separate preparations of mitochondria and the results averaged. All data are means and standard deviations.

The increased cellular respiration in sirtuin overexpressing cell lines was not associated with changes in cellular growth rate (Figure 2A), respiratory chain protein abundance (Figure 2B), or basal steady-state ATP levels. ATP levels were also measured after incubation with various metabolic inhibitors, including etomoxir (fatty acid oxidation inhibitor), oligomycin (ATP synthase inhibitor), and 2-deoxyglucose (glycolysis inhibitor). These experiments did not reveal any effect of sirtuin expression on the proportion of intracellular ATP being supplied by fatty acids versus glucose, or in the response to inhibiting oxidative phosphorylation (Figure 2C). Finally, because sirtuins consume NAD^+^ as part of their enzymatic reaction, we reasoned that constitutive sirtuin overexpression may increase the NADH/NAD ratio, thereby activating the respiratory chain [27]. Measuring NADH in isolated mitochondria proved difficult with highly variable results (not shown), likely due to the fact that much of the NADH in mitochondria is enzyme-bound [28]. Intramitochondrial NAD^+^, on the other hand, was measureable in mitochondrial extracts. Sirtuin overexpression caused a trend toward reduced NAD^+^ particularly in SIRT4 overexpressing cells, but these differences did not reach statistical significance (Figure 2D).

### Overexpression of mitochondrial sirtuins increases glycolysis

**Figure 3:**
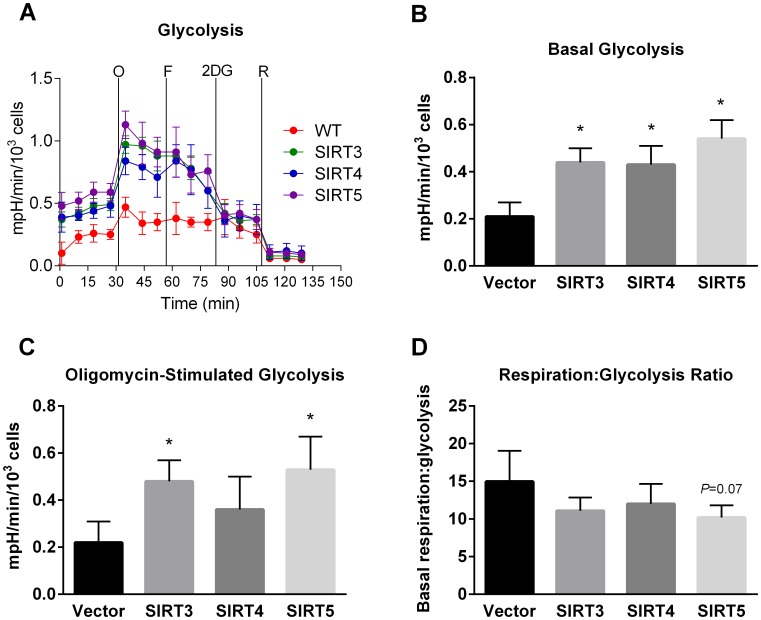
Effect of mitochondrial sirtuin expression on glycolysis. Seahorse extracellular acidification rates (A) were measured in quadruplicate wells containing equal numbers of cells. The experiment was repeated with similar results. Data collected over the first 30 minutes were averaged to yield the basal glycolytic rate (B). Oligomycin-stimulated glycolysis (C) was calculated by subtracting the basal values from the maximum values obtained immediately after oligomycin injection. The oxygen consumption/extracellular acidification ratio (D) was calculated by dividing the values shown in Figure 1D by those in Figure 3B. All graphs depict means and standard deviations, and *P<0.05. mpH = milli pH units.

In addition to oxidative phosphorylation, the Seahorse XF24 extracellular flux analyzer monitors the extracellular acidification rate which is an indicator of the glycolytic conversion of glucose to lactate. All three sirtuin overexpressing cell lines demonstrated significantly higher basal rates of glycolysis (Figure 3A, B). The glycolytic response to poisoning the electron transport chain with oligomycin was enhanced in SIRT3 and SIRT5 overexpressing cells but not SIRT4 overexpressing cells (Figure 3C). Finally, the ratio of basal oxygen consumption/extracellular acidification was calculated. This ratio is an index of the cells’ relative reliance upon mitochondria versus glycolysis for energy production. While there was a trend for reduced reliance upon oxidative phosphorylation relative to glycolysis for all three sirtuin-overexpressing cell lines, these trends did not reach statistical significance (Figure 3D).

### Overexpression of SIRT4, but not SIRT3 or SIRT5, alters mitochondrial superoxide and membrane potential

**Figure 4:**
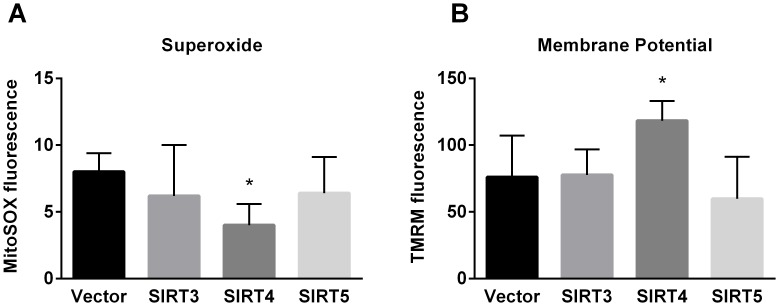
Effect of mitochondrial sirtuin expression on superoxide and mitochondrial membrane potential. (A) Superoxide measured with MitoSox. (B) Membrane potential measured with TMRM. Graphs depict means and standard deviations, and *P<0.05.

Superoxide levels and mitochondrial membrane potential are two parameters that are closely integrated with oxidative phosphorylation. Increased oxidative phosphorylation is often associated with increased levels of superoxide due to electron leakage from the respiratory chain [26]. Here, SIRT4 overexpression significantly reduced superoxide levels (Figure 4A). SIRT4 also significantly increased the mitochondrial membrane potential (Figure 4B). SIRT3 and SIRT5 overexpression did not alter these parameters.

### Overexpression of SIRT3, SIRT4, or SIRT5 increases mitochondrial substrate oxidation

**Figure 5:**
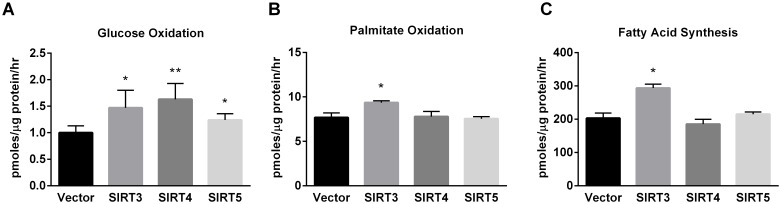
Effect of mitochondrial sirtuin expression on substrate flux. (A) Glucose oxidation to ^14^CO_2_. (B) ^3^H-palmitate oxidation to ^3^H_2_O. (C) ^14^C-acetate conversion to fatty acids. All graphs depict means and standard deviations, and *P<0.05.

The increases in both basal oxygen consumption and extracellular acidification would suggest increased energy metabolism in sirtuin overexpressing cell lines. Indeed, all three sirtuin-overexpressing cell lines exhibited increased rates of mitochondrial glucose oxidation to CO_2_ (Figure 5A), with SIRT4 having the greatest effect. In contrast, only SIRT3 increased fatty acid oxidation (Figure 5B), in keeping with its known role in the regulation of this pathway [29]. While fatty acid oxidation was increased in glucose-starved SIRT3 overexpressing cells (Figure 5B), SIRT3 overexpression in fed cells (complete DMEM with 25 mM glucose) was associated with a significant increase in fatty acid synthesis as measured by following the incorporation of radiolabeled acetate into intracellular lipids (Figure 5C).

### Physiological glucose normalizes bioenergetics in sirtuin-overexpressing cells

**Figure 6:**
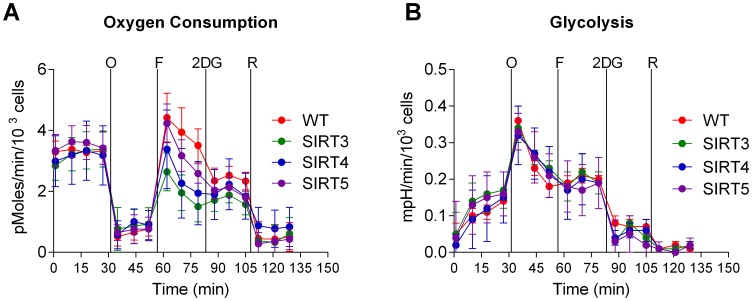
Seahorse XF24 extracellular flux analysis of sirtuin-expressing HEK293 cells under 5 mm glucose conditions. (A) Oxygen consumption and (B) extracellular acidification rates were measured under the same protocol as described in Figures 1 and 3. All graphs depict means and standard deviations. mpH = milli pH units.

All three sirtuin overexpressing cell lines exhibited increased glucose metabolism as evidenced by increased basal extracellular acidification and increased ^14^C-glucose oxidation to CO_2_. In a final set of experiments, the bioenergetics of these cell lines were re-examined following an overnight incubation in low-glucose media (5 mM), which more closely approximates physiological levels. Under these conditions the increase in basal oxygen consumption was abolished (Figure 6A) as was the increase in basal extracellular acidification (Figure 6B).

## Discussion

In the present work we have directly compared the bioenergetic consequences of SIRT3, SIRT4, and SIRT5 overexpression in the same cell type (HEK293). SIRT3 expression produced three HA-tagged isoforms, all localized to mitochondria, which confirms previous reports [24]. SIRT4-HA was also found only in mitochondria. SIRT5-HA localized to both mitochondria and cytosol, confirming a previous report [25]. In our hands, HEK293 cells expressed only a low level of endogenous SIRT3, and no endogenous SIRT4 or SIRT5 were detected. Thus, the relative degree of overexpression in our cell lines was much greater for SIRT4 and SIRT5, while the SIRT3 cell line approximately doubled the level of expression of the short isoform (28 kDa, or 30 kDa with HA tag), which is the best characterized isoform. This in essence would approximate the degree of SIRT3 28 kDa isoform induction that occurs *in vivo* under energetic stress such as fasting [29].

We observed that all three mitochondrial sirtuins can increase glycolysis, mitochondrial substrate oxidation, oxygen consumption, and mitochondrial proton leak in parallel, at least when glucose is abundant. However, we failed to provide evidence for a mechanism by which this occurs. We speculate that these shared effects of SIRT3, SIRT4, and SIRT5 overexpression may be due to driving down the NAD/NADH ratio by constitutive cleavage of NAD^+^. This would activate both the TCA cycle and the electron transport chain, which could trigger a coordinated increase in glucose utilization as evidenced by increased glycolysis and ^14^C-glucose conversion to ^14^CO_2_ [30], [31]. However, changes in the NAD/NADH ratio would not explain the observed increase in oligomycin-insensitive oxygen consumption which is thought to reflect proton leak [32]. Rather, we postulate that the increased proton leak may be due to increased activity of the ADP/ATP translocase, which has been shown to mediate two-thirds of the proton leak in most cell types [32]. SIRT3, SIRT4, and SIRT5 are all known to interact with ADP/ATP translocase [12], [13], [23], [33]. Increased proton leak through the ADP/ATP translocase is believed to suppress reactive oxygen species [32]. Indeed, despite higher oxygen consumption none of the sirtuin-overexpressing cell lines showed increased superoxide as measured by MitoSox fluorescence. SIRT3 has been shown to activate manganese superoxide dismutase which may also contribute to the lack of increase in superoxide in spite of increased respiration [34].

A review of the literature suggests that SIRT3 activates [4], [35], SIRT4 suppresses [15], [23], [36], [37], and SIRT5 can both activate and suppress mitochondrial function [10]–[12], [38], [39]. With regards to SIRT5, the majority of studies point to SIRT5 as being a positive regulator of mitochondrial function like SIRT3, with only one study observing SIRT5 as a mitochondrial suppressor [11]. Our present data support a role for SIRT5 as promoting mitochondrial function rather than repressing it. For SIRT4 our data are at direct odds with the literature. However, most of the data suggesting that SIRT4 represses metabolism and mitochondrial function comes from observations in the absence of SIRT4, i.e., SIRT4 knockout mice and cells. For example, in the absence of SIRT4, fatty acid oxidation and glutamine oxidation increase [15], [36], [37]. The induction of fatty acid oxidation is due to crosstalk with peroxisome proliferator-activated receptor-alpha and AMP-activated protein kinase, while the increase in glutamine metabolism is due to activation of glutamate dehydrogenase. Few studies have explored gain-of-function effects with SIRT4. In the study closest in design to ours, Ho et al [23] showed in HEK293T cells that SIRT4 overexpression reduces basal oxygen consumption. However, it is not clear from their report whether the SIRT4 overexpression was transient or stable, and SIRT4 protein levels were not presented. Further, oxygen consumption was measured in trypsinized, permeabilized cells in suspension (Ouraboros) rather than in a monolayer (Seahorse). Such methodological differences could account for the conflicting results. Moreover, HEK293T cells, which are derived from HEK293 cells and engineered to stably express the SV40 large T antigen, have been reported to be predominantly glycolytic when measured in a Seahorse extracellular flux analyzer, with oxygen consumption/extracellular acidification ratios around 3 in 25 mM glucose [40]. In comparison, our HEK293 cells show ratios in the range of 10–15 in 25 mM glucose and this doubles under 5 mM glucose (data not shown). Overexpression of SIRT4 in a predominantly glycolytic cell line may produce different results than overexpression in a cell line that is more reliant upon mitochondria for energy metabolism. In short, the conflicting results between the present work and that of Ho et al [23] highlights an important caveat of both studies, which is that while cell culture models are convenient for manipulating and measuring metabolic parameters, the data must be interpreted with caution as many variables may contribute to how a given cell line responds to perturbation.

In our experiments the effect of SIRT3, SIRT4, and SIRT5 on glycolysis and oxygen consumption disappeared when the glucose concentration was reduced. This observation may have implications for the role of these sirtuins in pathophysiological states such as cancer and diabetes. Under high glucose SIRT3, SIRT4, and SIRT5 may have an anti-Warburg effect by stimulating oxygen consumption in parallel with the increased glycolysis that typically accompanies elevated glucose. The excess energy consumed is dissipated by proton leak across the mitochondrial inner membrane rather than being channeled into increased growth or proliferation. The proton leak further protects against the accumulation of reactive oxygen species. This may be part of the mechanism behind SIRT3, SIRT4, and SIRT5 appearing to have tumor-suppressive effects [37], [38], [41]. Further work is underway to investigate the nutrient sensing roles of SIRT3, SIRT4, and SIRT5 under normal and pathological conditions.
